# Large-Scale Deployment of Lehmann’s Funnel Entry Traps to Control Malaria Mosquito Populations

**DOI:** 10.3390/tropicalmed10020049

**Published:** 2025-02-07

**Authors:** Hamidou Maïga, Roger Sanou, Bazoumana B. D. Sow, Adama Ouema, Abdoul Azize Millogo, Koama Bayili, Aristide Sawdetuo Hien, Simon P. Sawadogo, Souro Abel Millogo, Adrien Marie Gaston Belem, Léa Paré Toé, Roch K. Dabiré, Abdoulaye Diabaté

**Affiliations:** 1 Institut de Recherche en Sciences de la Santé (IRSS)/Direction Régionale de l’Ouest (IRSS-DRO), Bobo-Dioulasso 01 BP 545, Burkina Faso; sanourog@yahoo.fr (R.S.); sowbazoumana@gmail.com (B.B.D.S.); ouemaadama@gmail.com (A.O.); azizemillogo@gmail.com (A.A.M.); kwamajacques@yahoo.fr (K.B.); aristide.hien@yahoo.fr (A.S.H.); sawsimp2005@yahoo.fr (S.P.S.); milabso@yahoo.fr (S.A.M.); lea.paretoe@gmail.com (L.P.T.); dabireroch@gmail.com (R.K.D.); npiediab@gmail.com (A.D.); 2 Centre Muraz, Institut National de Santé Publique (INSP), Bobo-Dioulasso P.O. Box 390, Burkina Faso; 3 Université Nazi BONI, Bobo-Dioulasso P.O. Box 1091, Burkina Faso; belemamg@hotmail.fr

**Keywords:** malaria, vectors, trapping, elimination, density

## Abstract

Effective tools to prevent mosquito bites are essential for malaria control. The Lehmann Funnel Entry Trap (LFET), a window screen proven effective in reducing mosquito density, was tested for its large-scale impact on malaria vector control and community acceptance. A total of 1313 traps were deployed in Vallée du Kou 3 (VK3), with 12 traps randomly selected for detailed evaluation against untrapped houses in Vallée du Kou 5 (VK5). Traps were placed in windows with doors blocked by curtains. From July to October, mosquitoes were collected for nine days per month from VK3 traps and VK5 control houses. Morphological identification, density analysis, resistance gene screening, and female age structure determination were conducted. The trap’s impact was assessed via pyrethrum spray catch (PSC) and for nine days per month, while trap fabric integrity and community perceptions were also evaluated. Traps reduced mosquito entry density by more than 90% in VK3 houses. VK3 had 33% higher house mosquito density pre-intervention and 47% lower house mosquito density post-intervention than VK5. Old female mosquito numbers rose in VK5 but not VK3. Pyrethroid resistance was high (kdr mutation frequency > 0.9) in both control and intervention sites. VK3 residents appreciated the traps for reducing bites and improving sleep. The LFET effectively controls malaria vectors and is well-suited for widespread use in malaria elimination efforts.

## 1. Introduction

Vector control has contributed to the reduction in malaria burden over the last few years. According to a recent WHO report, the number of deaths due to malaria in 2023 is estimated to be 597,000 [1]. Vector control such as Indoor Residual Spraying (IRS) and the mass distribution campaigns of Insecticide Treated Nets (ITNs) have contributed to malaria reduction [2]. However, the emergence and spread of insecticide resistance in malaria vectors is threatening the future of vector control [3,4,5] in WHO African region, making it clear that additional tools are needed to sustain vector control toward malaria elimination. Thus, discovering new tools or improving the effectiveness of the existing ones should be prioritized [3].

Since the link between malaria and mosquitoes was established, window screening and nets have been used as part of malaria vector control management [6,7]. More recently, studies using screens within windows, on ceilings, and on doors have proven to be effective in reducing the number of mosquitoes entering homes. Consequently, there is added personal protection against mosquito bites as well as community protection through added mosquito mortality [8,9,10,11]. Whilst some studies have established a relation between malaria illness and the type and quality of dwelling construction [12,13], other studies have demonstrated that in addition to quality of construction of the home, modifications to prevent mosquitoes from entering houses can help reduce malaria transmission by lowering human exposure to infectious bites [7,10,11,14,15,16,17,18]. Interestingly, the use of mosquito-repellent plants in the home has also been shown to reduce mosquito densities between 56 and 83% [19], and there is a significant reduction in anemia (19% vs. 12%) in children living in homes with window screens [16,20].

Although these methods deny mosquitoes’ entry to a dwelling, the mosquitoes are not killed. So, they can still bite outdoors and transmit malaria parasites [21,22,23,24,25,26,27]. As such, denying mosquito access to indoor living spaces is a worthy cause, but killing blood-seeking (and resting-site-seeking) mosquitoes could be a more effective goal.

This proposal led to the development of a new approach, which considers mosquito behavior and typical house entry routes for mosquitoes. Within this approach, Lehmann Funnel Entry traps (LFETs) were tested in high and low vector density settings as a malaria vector control tool in development [9]. Recently, three new prototypes were created and successfully tested in two ecological settings to the southwest and northwest of Bobo-Dioulasso, Burkina Faso [28], in view of further use against malaria vectors. Once installed into a window, a LFET catches and kills all mosquitoes that attempt to access the house through the relevant entry point. Consequently, household members may be prevented from being bitten if all windows are secured with traps. Once a mosquito enters the small opening of the trap outside the home, it becomes trapped and will remain so until dehydration kills it. We hypothesized that the use of entry traps being fixed on the windows will reduce indoor entry mosquito density. Therefore, blocking windows within a house with screens will help with removing indoor resting and anthropophagic mosquitoes.

Most vector control interventions against malaria require active participation from the local population to ensure proper adherence [29,30]. Recent studies have demonstrated the important role of stakeholder engagement in the successful control and elimination of many infectious diseases. Communities should receive comprehensive details about the intervention or program right from the start of any study, ideally during the program’s design phase, and continue to be informed throughout and at its conclusion. An accurate timeline is also important for transparency [31,32,33]. Furthermore, as the space within the household has multiple functions, the homeowner’s full buy-in is required, as LFETs (or any other materials important to the study) may require reorganization at home. Given that the LFET is an indoor intervention tool, community engagement is especially important to ensure the traps are sustainable and fully operational throughout the use period.

The objectives of this trial were to assess the impact of a large-scale deployment of traps in terms of indoor mosquito density reduction in an intervention village compared to a control village. In addition, the age of mosquitoes, as well as the physical integrity of the traps’ nets, were assessed over the study period. Sociological endpoints were also reported in this study.

## 2. Materials and Methods

### 2.1. Study Area and General Household Census in VK3

**Figure 1:**
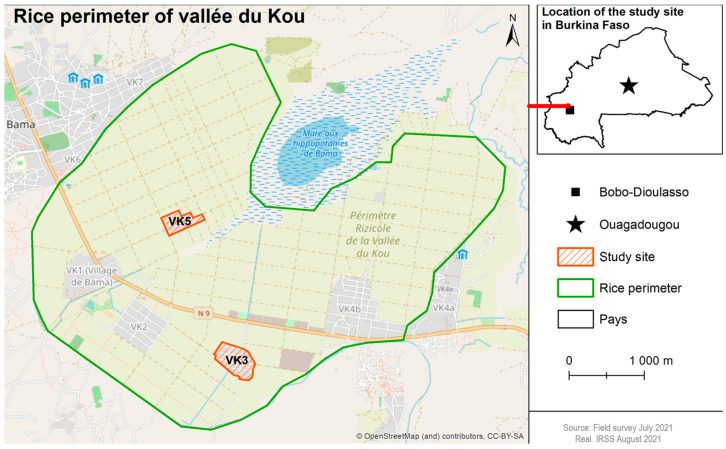
Map of Vallée du Kou (VK) and its villages (VK1, 2, 3, 4, 5, 6, and 7).

The study was carried out in Vallée du Kou, Burkina Faso (11°23′ N, 4°24′ W), in an irrigated rice field area. The site is characterized by wooded savannah across 1200 ha and contains seven discrete villages. Relatively high mosquito densities are observed annually during August and September, corresponding to the peak of the rainy season. *Anopheles coluzzii* is predominant throughout the year and *An. gambiae* is observed toward the end of the rainy season (frequencies fluctuating between 5% and 20%). Both species are highly resistant to pyrethroids and DDT (kdr frequency (0.8–0.95) [9,34,35,36]. Two villages, Vallée du Kou 3 (VK3) and Vallée du Kou 5 (VK5), were selected as intervention village (IV) and control village (CV), respectively (Figure 1).

These two villages were selected because they had similar ecology in terms of mosquito densities and species [37] and were situated 1 km apart.

**Figure 2:**
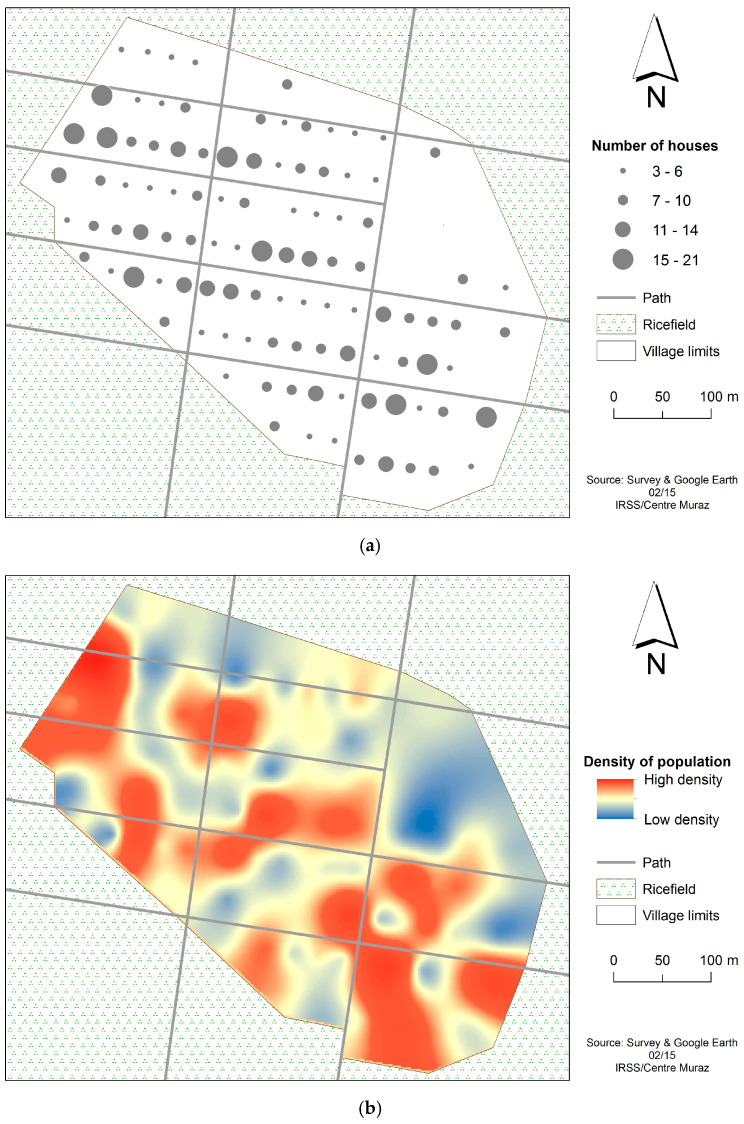
(**a**) Georeferencing of houses/windows distribution in VK3, (**b**) the human density related to the number of houses (from 3 up to 21). The number of houses ranged from 3 to 21. For an average of 5–10 people per house, the low-density population (blue) ranged from 15 to 30 persons, while the high-density population (red) may have up to 105–210 persons. A scale of 0–100 m was considered. The bigger the dots, the higher the population density. This is relative to the house density where the larger human population leaves.

A general survey of the houses and windows was conducted in VK3 using a Global Positioning System (GPS) [38] to count all the inhabited houses (Figure 2a,b). A scale of 0–100 m was considered. The bigger the dots, the higher the population density. This is relative to the house density where the larger human population leaves.

### 2.2. Study Design and Period

The study was carried out in two sequential phases that encapsulated trap manufacturing and large-scale field deployment of the Lehmann Funnel Entry Trap.

#### 2.2.1. Trap Manufacturing

**Figure 3:**
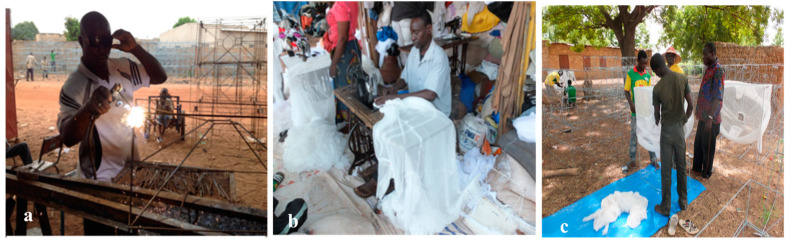
(**a**) Local window manufacturer, (**b**) tailor sewing traps, and (**c**) local field entomology workers recruited to install traps.

The traps were made from a metal frame and were fitted from the bottom to the top with a mosquito net to prevent any mosquitoes or other insects from escaping the trap once inside (see Sanou et al. [28] for more details). The metal manufacturer first produced a sample of each trap size (Figure 3a).

The trap samples were also sent to a tailor for the nets to be made to size (Figure 3b). Then, the remaining traps were produced, painted with neutral oil, and labeled according to their dimensions, before transportation to VK3. Nets were then mounted on the trap metal frames (Figure 3c) before installation. Each manufactured net covers the trap entirely, which fits into the window perfectly. Each trap’s net also has a sleeve for easy access, opening and/or closing the window.

All the windows of the inhabited houses were counted and measured to manufacture the traps accordingly.

#### 2.2.2. Large-Scale Field Evaluation of the Lehmann Funnel Entry Trap

##### Deployment and Installation of the Traps in VK3

For trap deployment/installation, all eaves and holes in the houses were blocked using cloth or sponge and a curtain was placed at each door by a large team of local and technical workers. Non-inhabited house windows were secured simply with a net, to reduce the number of mosquito-resting sites.

**Figure 4:**
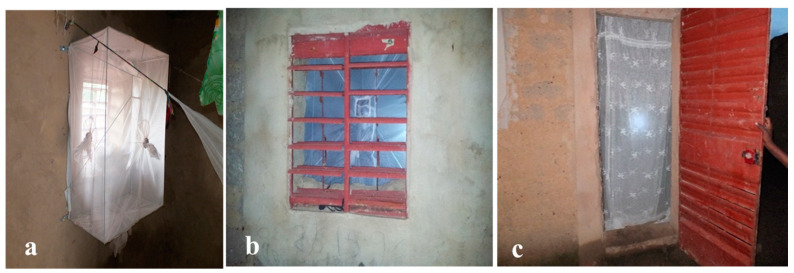
(**a**) Lehmann Funnel Entry Trap within a house, (**b**) outward view through window, (**c**) a curtain at a door.

In total, 1313 traps were placed within the windows of houses to intercept incoming mosquitoes (Figure 4a,b). A new curtain made from regular cloth was provided to each house in VK3 to block mosquitoes from entering through the door (Figure 4c). No constraints were required on the use of the doors or windows, and occupants were free to go to bed at any time.

##### Mosquito Collection

To assess the trap performance, a mosquito collection was performed for nine days per month from 12 selected traps (trapped mosquitoes) and the same houses as LFETs (indoor resting mosquitoes) in VK3, while only indoor resting mosquitoes from eight houses were collected in VK5. The traps were emptied the day before collection so that the numbers reflect the amount entering on a single night. Single room houses (a single house with one window and one door) were randomly selected according to their geographic location (central, north, east, west, and south) in both villages. Houses were located far from each other, spaced at least 10–50 m apart to avoid human attractivity bias, and were monitored over nine days per month in both villages from July to October. Mosquitoes were manually collected (on 36 collection days over the four months of the trial) with mouth aspirators in the traps and same house as LFET (for two hours) by three experienced collectors. The mosquito collection was simultaneously carried out in VK3 and VK5.

To provide evidence of the impact of the traps being deployed on indoor mosquito density reduction, a pyrethrum spray catch (PSC) [39] was carried out simultaneously one day per month over the three-month trial, in 10 randomly selected houses in each village. These houses were different to the regular study houses in the villages.

##### Assessment of Mosquito Species Identification and Allelic Frequencies of kdr Mutation

Collected mosquitoes from traps and houses (VK3) and indoor mosquitoes from VK5 were morphologically identified to the genus level, and assessed for physiological status [40], under a microscope. They were killed using chloroform before being counted. A sub-sample was then preserved in 80% ethanol vials for subsequent genotyping to species level and to check on the frequency of the knockdown resistance (kdr) mutation using previously published protocols [41,42].

##### Assessing Wild Female Mosquito Parity During Trap Deployment

In addition, to assess the mosquito population age structure from July to October, around 55 unfed female *An. gambiae* collected from the traps and houses were dissected and classified into parous and nulliparous mosquitoes, according to Detinova’s protocol [43].

##### Physical Conditions and Cleanliness of the Traps During the Trial

After trap installation, 50 nets were sampled and were checked for their physical integrity in 50 selected households over two months (one and two months after installation). The assessment of the integrity of the fabric was performed by visual examination without removing the nets. Any holes observed were assigned to one of four size categories according to WHO guidelines [44]: a hole size of 0.5–2.0 cm or ‘< a thumb-sized opening’; a hole size of 2.0–10.0 cm or ‘> a thumb but < a fist’; a hole size of 10–25 cm or ‘> a fist but < a head’; and a hole size of > 2.5 cm or ‘> a head’.

General trap integrity was assessed based on two measurements such as the proportion of nets with any observed hole(s) and the proportionate holes index (pHI) for each net. The proportion of nets with any observed hole(s) was determined by counting the number of tears and holes as described: total number of coded nets with at least one hole of the size (1 − 4) × 100/total number of nets assessed in surveyed households. The pHI for each net was calculated as the sum of the holes weighed by size for each net. For this group, the weights used to calculate the pHI were 1, 23, 196, and 576 as described below: pHI = 1 × number of size − 1 holes + 23 × number of size − 2 holes + 196 × number of size − 3 holes + 576 × number of size − 4 holes. To better correlate the holes index to an integrity status (net condition) for each sampled net, the pHI was categorized into ‘good’ (pHI ≤ 64), ‘serviceable’ (pHI ≤ 768) and ‘replace’ (pHI > 768).

The trap net dirtiness (/cleanness) was also evaluated, and nets were classified and categorized into ‘clean’, ‘a bit dirty’, ‘dirty’, and ‘very dirty’. When the net was deemed irreparably damaged by dirtiness, it was replaced by another net of the same size.

##### Socio-Anthropological Investigation on the Use of the Traps

Qualitative and quantitative surveys were conducted from March to August from the beneficiaries of the LFETs in VK3. The qualitative survey consisted of individual interviews with members of the community about their perception of the traps. There were direct observations of traps being used in the village. The usage of traps was witnessed elsewhere. The quantitative survey covered 276 inhabitants and was based on the level of acceptance of the trap by its users, the trap’s perceived effectiveness, and the limitations of the trap.

In addition, all inconveniences reported by users of the traps were recorded by a social worker and reported for subsequent remedial measures. A follow-up survey according to the WHO indices [44] was conducted once the traps were installed, any mishandling was also reported, and action was taken to resolve any problems raised either by a social worker or the users themselves.

##### Parameters Measured and Statistical Analysis

The reduction in indoor mosquito density per house was calculated as follows:

Reduction = (Number of mosquitoes caught in the trap)/(Total number of mosquitoes),

where Total number of mosquitoes = Number of mosquitoes caught in the trap + Number of mosquitoes in the same house as LFET. Direct mortality was estimated as the number of dead mosquitoes caught in the traps out of collected mosquitoes from the trap. The allele frequency was calculated as the number of resistant mosquito homozygote resistant (2nRR) and hybrid resistant (nRS) out of the 2× total of homozygote resistant (RR), and susceptible (SS), and RS the hybrid resistant–susceptible. Mosquito age structure (parity) was defined as the number of female mosquitoes that laid eggs (parous) and nulliparous out of a total of dissected mosquitoes. The physical integrity of the traps to check how a trap’s net was maintained in the intervention village, was analyzed using descriptive statistics (mean, median, interquartile range) to compare pHI values. The socio-anthropological investigation (acceptability) on the use of traps by end-users and calculated by counting the “Yes” or “No” answers to the relevant questions, relating to the total number of interviewees.

Descriptive data were summarized, inputted, and cross-checked in Microsoft Excel 2007 (Microsoft^®^, New York, NY, USA), and R-4.0.4 was used to produce tables, graphs, counts, means and standard errors. A Generalized Linear Mixed Model (GLMM) with a Poisson or negative binomial distribution was used to choose the suitable distribution of the mosquitoes collected in the traps and houses from VK3 and VK5, respectively. A zero-inflated Poisson mixed regression modeling tool was used to estimate the intervention (trap) effect on daily numbers of mosquitoes collected, while accounting for a possible spatial variation in terms of total mosquitoes collected between VK5 and VK3. This was to check for a possible difference in terms of physiological status (gravid, blood fed, unfed). To assess the dynamic of mosquitoes between VK3 and VK5, pyrethrum spray catch data were analyzed using a non-parametric pairwise test, Anova-glmmTMB. The month of mosquito collection was used as a random effect to account for temporal variability. A one-way Anova was used to compare the age distribution of mosquitoes between VK3 and VK5.

## 3. Results

### 3.1. Large-Scale Field Evaluation of the Lehmann Funnel Entry Trap

#### 3.1.1. Number of Anopheles Mosquitoes Trapped and Their Feeding Status

A total of 18,884 and 15,650 *An. gambiae* mosquitoes were collected over the study period in VK3 (from selected traps and matched houses) and in VK5 (inside houses), respectively. Out of this, 87% (16,437 of the 18,884) were collected from the traps, including the following live mosquitoes: unfed (49%), blood-fed (40%), and gravid females (8%); and the following dead mosquitoes: unfed (1%), blood-fed (1%) and gravid females (1%). This compared to 13% (2447 of the 18,884) from the same house as LFET in VK3. In VK5, of the 15,650 mosquitoes collected, 67% were found unfed, 28% blood-fed and 5% were gravid females.

The sampling on average per night per trap in VK3 for dead mosquitoes ranged from 0.95 ± 0.19 to 31.76 ± 4.40 (means ± se), and for live mosquitoes from 8.96 ± 0.92 to 54.99 ± 5.34, caught in a trap in VK3. This sampling average varied from 2.77 ± 0.43 to 10.18 ± 2.12 for live mosquitoes collected indoor in VK3, compared to between 1.65 ± 0.18 and 21.44 ± 2.02 for live mosquitoes collected indoors in VK5 (Table 1, Figure S1). No dead mosquitoes were collected from the houses in either site.

There were significantly fewer mosquitoes collected from the houses in VK3 as compared to VK5, regardless of the gonotrophic status (unfed, df = 0.4964, *χ*^2^ = 2175, *p* < 0.0001, blood-fed, df = 0.3525, *χ*^2^ = 1193.2, *p* < 0.0001, and gravid, df = 0.1439, *χ*^2^ = 665.27, *p* < 0.0001) (Table S1).

#### 3.1.2. Number of Other Mosquito Species Trapped

During the study, 1169 other mosquitoes were caught in traps, including species as *An. coustani* (2.6%), *An. pharoensis* (6.0%), *Culex sp* (87.3%), *Mansonia sp* (4.1%), and *Aedes* (0%) in VK3. In addition, 89 mosquitoes were collected in matched houses and identified as *An. coustani* (2.6%), *An. pharoensis* (6.7%), *Culex sp* (87.6%), *Mansonia sp* (1.1%).

In VK5, 707 mosquitoes were collected from the houses comprising *An. coustani* (0.4%), *An. pharoensis* (4.2%), *An. rufipes* (0.1%), *Culex sp* (87.7%), and *Mansonia sp* (0.4%) (Table S2).

#### 3.1.3. Assessment of the Mosquito Density Reduction over the Three Months of Study in VK3 and VK5

**Figure 5:**
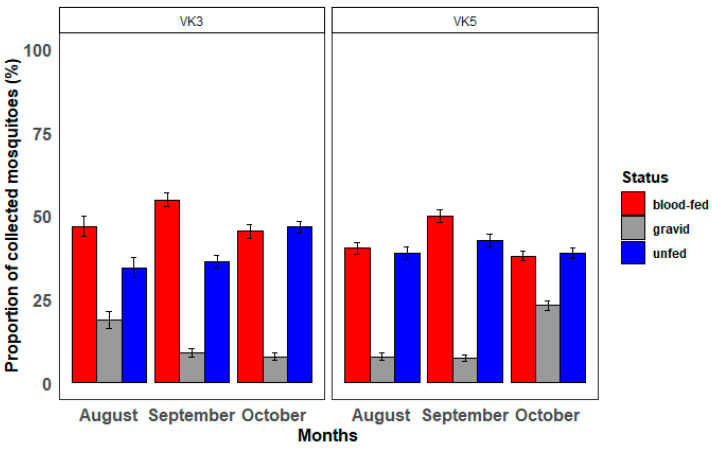
Proportion of collected indoor resting mosquitoes in Vallee du Kou (VK3 vs. VK5) per month (August, September, October) according to their feeding status (blood-fed, gravid, unfed).

The proportion of indoor resting mosquitoes collected using PSC, showed an increase in the proportion of unfed females and a decline in the proportion of gravid females in VK3, while in VK5, there was a similar trend in the proportion of unfed and an increase in gravid females (Figure 5).

Overall, the estimated reduction rate using PSC, was higher in VK3 (97.3 ± 11.01%, 95% CI: 77.8–121.7%) compared to VK5 (64.4 ± 7.31%, 95% CI: 51.5–80.6%) (indoor reduction rate VK5/VK3 = 0.662 se = 0.0113, df = 176, t-ratio = −24.131, *p* < 0.0001) over three consecutive months (from August to October) (Table S3).

### 3.2. Assessment of Mosquito Species Identification and Allelic Frequency of kdr Mutation

*Anopheles coluzzii* was identified as the only *Anopheles* species in VK3 and VK5, regardless of where mosquitoes were collected (trap, indoor). The kdr mutation was genotyped in mosquitoes to estimate their frequencies in the natural populations from Vallée du Kou. Out of 512 sub-samples analyzed, PCR assay revealed that most of the mosquitoes were highly resistant to pyrethroids and DDT (kdr mutation frequency: trap: 0.96, indoor: 0.93).

### 3.3. Assessing Wild Female Mosquito Parity During Trap Deployment

Ovary dissection indicated that the mosquito population kept a stable age structure as compared to the beginning of the intervention with ~2% (1/55) of the population being parous females in both VK3 and VK5 (*χ*^2^ = 0.00016532, df = 1, *p*-value = 0.9897). Toward the end of the intervention, 12% (7/58) of the females were parous in VK5 as compared to ~3% (2/69) in VK3 (*χ*^2^ = 4.0247, df = 1, *p*-value = 0.04484) (Table S4).

### 3.4. Physical Condition and Cleanliness of the Traps During the Trial

The proportion of nets with at least one hole was ~76% over the two months of the study. Moreover, the number of holes (141) was higher in the second month of the survey than in the first month (91). The mean score of the Hole index was significantly higher in the second month at 2.86 (95% CI = 2.079–3.641) as compared to the first month with 1.82 (95% CI = 1.244–2.396; *p* = 0.0298). The number of holes did not seem to change significantly based on the location of the net, with 69 holes found on entry window nets and 74 holes on rear window nets (*p* > 0.05). The data showed a greater number of holes in category hole-1 than that observed in the two other groups (categories hole-2 and hole-3) (121:20:1, respectively) (*p* < 0.0001). The location of holes in the net was explored during the second month. Of the 141 counted holes, ~53 were found on the front of the nets, 38 on the seams, 30 on the length, and 20 on the width.

Twenty five percent of the holes were found along the length and width of the net, followed by 10% and less than 1%, respectively, on the front side and the seams of the net. The median proportionate hole index (pHI) was 3.5 with IQR (2–27.5). The pHI for each net showed a utilization rate of 66% (33/50) of nets found in good condition (pHI ≤ 64) and only 1% was found to be just serviceable (pHI ≤ 768—serviceable). About 75% of the nets were deemed to be clean. The dirty nets were typically installed at the windows of houses built with bricks made of mud. Moreover, the dirtiest nets were located at the front of the house. At the end of each survey, nets with several holes were replaced by new ones with the same dimensions.

### 3.5. Socio-Anthropological Investigation on the Use of the Traps: Acceptance and Benefits Attached to the Trap

Qualitative and quantitative data showed a good level of acceptance of the traps by the interviewees. About 80.8% (223/276) of the beneficiaries declared that the trap reduced the number of mosquitoes in the house. This was appreciated by the users, as shown by the following quote, which is taken from an interview with a resident: “when you look into the object, there is a lot of mosquitoes like that. If all these mosquitoes should enter the home! Good gracious!” Table 2 shows the details of the various benefits that the traps brought to the population of VK3.

Importantly, 98.55% (272/276) of the respondents claim to have had peaceful sleep since trap installation, and 98.91% believe that the trap will help reduce disease in the area.

## 4. Discussion

The main objective of this study was to show the potential of the LFET to control malaria vectors in a rice-growing and high mosquito population settings. In Burkina Faso, most mosquitoes are resistant to at least one class of insecticides used in public health, imposing research focused on finding alternative solutions is necessary to sustain the results gained in the fight against malaria. Here, we evaluated the novel trap, the LFET at a village scale. A total of 1313 traps were deployed in VK3, which contributed to a reduction in indoor mosquito density of more than 90% as compared to VK5 (control site) and a good level of acceptance of the traps, throughout the study period. The LFETs have proven to be effective in suppressing *An. gambiae* species and have caught various other mosquito species, including species that might have bitten the villagers if no trap had been deployed. Traps protected the population of the village from indoor mosquito biting in addition to the other malaria control methods already in use such as bed nets, IRS, repellent plants, etc. The findings of this study align with previous research indicating that screening decreased the entry of malaria-carrying mosquitoes into homes and helped prevent anemia in children [16,20]. House screening, particularly of eaves, doors, and windows, has shown significant promise in decreasing indoor mosquito densities and malaria transmission [17,45]. Push-pull tactics, involving repellents and attractant-baited traps, have also been investigated, with eave screening reducing house entry by malaria vectors [45]. Although no epidemiological data were collected during our trial, the use of the LFET could have consequently reduced malaria transmission in the village.

Ensuring complete trap coverage in VK3 may have contributed to indoor mosquito density reduction as mosquitoes were repeatedly trapped after emerging from rice breeding sites. In addition, more blood-fed mosquitoes were caught overtime during this trial, probably looking for a resting site to digest blood. Trapping these blood-fed mosquitoes could, to a lesser extent, reduce the number of mosquitoes in the laying process and ultimately reduce the number of mosquitoes in the trial setting. However, as the intervention village is surrounded by water, it would be challenging to reduce mosquito density to a very low level. This challenge might have been somewhat reduced if the study site had been located in remote villages away from water sources. In such cases, the suppressive effect of the trap would likely have been clearer.

The successful implementation of LFET installation as a malaria tool may focus on the direct monitoring of the adult mosquito population, as this provides a real-time picture of the effects of the intervention [46]. As part of this direct monitoring, the PSC results indicated the effectiveness of traps in removing mosquitoes from the environment. In VK3, mosquitoes entered houses and were not able to get out, whereas in VK5, there were no traps and so the mosquito population density remained more and less constant.

In this study, the mosquito population was exclusively composed of *An. coluzzii* in Vallée du Kou, which aligns with the previous studies [28,46]. The kdr L1014F mutation conferring resistance to pyrethroids and to DDT was high (>0.9) when the study was conducted. This result is consistent with previous studies reporting a high level of resistance in mosquito populations due to the extensive use of pesticides in rice and cotton fields in the village [34,46]. The LFET has proven efficacy in controlling highly resistant malaria vectors. LFETs were set up in VK3, contributing to controlling mosquito entry into houses, as most mosquitoes collected from the traps were blood-fed, gravid, or unfed. Without these traps, the mosquitoes would have entered to rest or seek human blood indoors. Interestingly, installing traps enhanced the protection offered by existing tools such as LLINs and IRS inside houses, reducing mosquito bites and ultimately the risk of malaria. Significant efforts should be made to reduce insecticide pressure in malaria control tools such as LFETs to help not only control but also mitigate the increase in insecticide resistance. Importantly, no insecticides were used on LFETs, aiming to lessen the existing insecticide pressure in the area, which is already high due to pesticide use in rice and cotton cultivation around the trial site.

Interestingly, the dissection of the ovaries of collected mosquitoes indicated that the mosquito population had the same age structure at the beginning of the intervention (~2% female parous) in both villages. However, toward the end of the intervention, 12% of the dissected females were scored parous in the control village against ~3% in the intervention village, indicating that the trap was cumulatively extracting and killing old females capable of transmitting malaria in the intervention village. Moreover, traps removed and killed a proportion of older outdoor mosquitoes. These outdoor mosquitoes may have impacted outdoor malaria transmission, as shown in other studies [24,47].

VK3 is one of seven discrete villages (VK1 to VK7) within the Vallée du Kou area. The village is close to its sister villages VK2 and VK4, on the western and eastern side, respectively, and is surrounded by rice fields. The poor isolation of VK3 has contributed to its sustained high mosquito density. It has been shown that mosquitoes can disperse up to two kilometers looking for a blood meal, so this could have impacted mosquito density in VK3 [48].

A study completed in the same ecological setting in Burkina Faso has shown that newer branded nets had a lower efficacy, which makes it clear that additional intervention tools are needed to control resistant mosquito populations [49]. The fact that the trap can help to control the mosquito population makes it very attractive. Once installed, the trap requires minimal adaptations in human behavior, and unlike mosquito bite prevention tools such as bednets, it protects everyone sleeping within the dwelling. Importantly, most interviewees acknowledged that the trap reduced mosquito biting and allowed them to sleep peacefully, which is one of the most important criteria that impacts the acceptability of conventional intervention tools such as bed nets [50]. The high acceptance of the LFET may explain the overall good condition of the traps seen at one to two months post-deployment.

Although the retail price of a single trap was estimated to be too high by the villagers, it is worth noting that they all wanted to keep the trap after the evaluation, meaning that the trap was very useful to them. Commonly, most households in Burkina Faso receive malaria preventive methods, including bed nets, for free [51]. A cost-effectiveness study has not yet been performed, but it is important to note that a single trap installed in a house protects everyone sleeping.

## 5. Conclusions

The LFET has proven efficacy in controlling highly resistant malaria vectors. The acceptance of the trap was high, as the beneficiaries could see the number of mosquitoes trapped in it every day, and it was acknowledged that the trap reduced mosquito biting and allowed peaceful sleep. Although the deployment of the LFET across an entire village showed a positive impact, a trial in an isolated site with a high mosquito population could better show the suppressing effect of the trap. Additionally, a randomized controlled study design could better show the effects of the traps on mosquito density reduction. No survey data on parasitemia and malaria cases were collected during this trial. So, a trial with malaria epidemiological endpoints could link the performance of the trap against malaria. Nevertheless, growing evidence shows that the trap installation in VK3 may have reduced malaria transmission [52].

Assuming that the trap size can be further reduced and the price to the user can be lowered, LFETs may represent a viable business opportunity to entrepreneurs. The next step is to build a business plan and work alongside local window manufacturers to help them to start manufacturing the traps on a large scale. Efforts will be devoted toward the national malaria control program in Burkina Faso to integrate the LFET into the malaria control toolbox.

## Figures and Tables

**Table 1:** Descriptive table of gonotrophic status of *Anopheles gambiae* female mosquitoes in VK3 and VK5 over the study period.

Village	Collection Method	Status	Gonotrophic Status	Total	% Status	Average	Std_error	Median	Maximum
VK5	house	alive	unfed	10,461	67	21.44	2.02	4	436
			blood-fed	4384	28	8.98	1	1	224
			gravid	805	5	1.65	0.18	0	53
VK3	house	alive	unfed	421	17	2.77	0.43	1	36
			blood-fed	1548	63	10.18	2.12	3	197
			gravid	478	20	3.14	0.59	1	69
	trap	alive	unfed	7973	49	54.99	5.34	31	283
			blood-fed	6654	40	43.78	3.9	28	261
			gravid	1362	8	8.96	0.92	5	69
		dead	unfed	144	1	31.76	4.4	296	31.76
			blood-fed	152	1	6.22	1.21	126	6.22
			gravid	152	1	0.95	0.19	18	0.95

Std = standard; VK3 = Vallee du Kou, sector 3, VK5 = Vallee du Kou, sector 5.

**Table 2:** Socio-anthropological investigation on the use of the Lehmann Funnel Entry Traps over the study period in VK3, Acceptance and benefits attached to the trap.

Answers/Modalities	% YES (n)	% NO (n)
Reduction in mosquito bites	85.40 (223)	14.60 (56)
Reduction in mosquito noise	31.80 (88)	68.00 (188)
Reduction in disease	98.50 (272)	1.00 (4)
Reduction in malaria	98.91 (273)	1.00 (3)
Peaceful sleep	93.84 (259)	6.00 (17)
Protection against other insects	77.54 (274)	18.00 (62)
Preventing mosquitoes from entering the house	52.17 (144)	48.00 (132)

## Data Availability

The data supporting the findings of this study are available upon reasonable request.

## Supplementary Materials

The following supporting information can be downloaded at: https://www.mdpi.com/article/10.3390/tropicalmed10020049/s1, Figure S1. *Anopheles gambiae* females gonotrophic status per day of mosquito catch (a) in both VK3 and VK5 villages and (b) summary of catches over the study period. Table S1. Comparison and proportions of *Anopheles gambiae* female mosquitoes collected per house in VK3 and VK5 over the study period; Table S2. Numbers and proportions of other mosquito species caught in trap and house in VK3 and VK5 over the study period; Table S3. Comparison of the mosquito density reduction over three months of the study period in VK3 and VK5; Table S4. Assessing wild female mosquito parity during trap deployment in July, August and October.

## Abbreviations

The following abbreviations are used in this manuscript:

VK3	Vallée du Kou 3
VK5	Vallée du Kou 5
GLMM	Generalized Linear Mixed Models
LFET	Lehmann Funnel Entry Trap
