# Supplementary material for: Large-Scale Deployment of Lehmann’s Funnel Entry Traps to Control Malaria Mosquito Populations
Source: Trop Med Infect Dis. 2025 Feb 7;10(2):49. doi: 10.3390/tropicalmed10020049 (PMC11861795; doi:10.3390/tropicalmed10020049)
Supplement: Supplementary file 1 [file tropicalmed-10-00049-s001.zip › LFET2_Supplementary_Figure S1.pptx]

## Slide 1
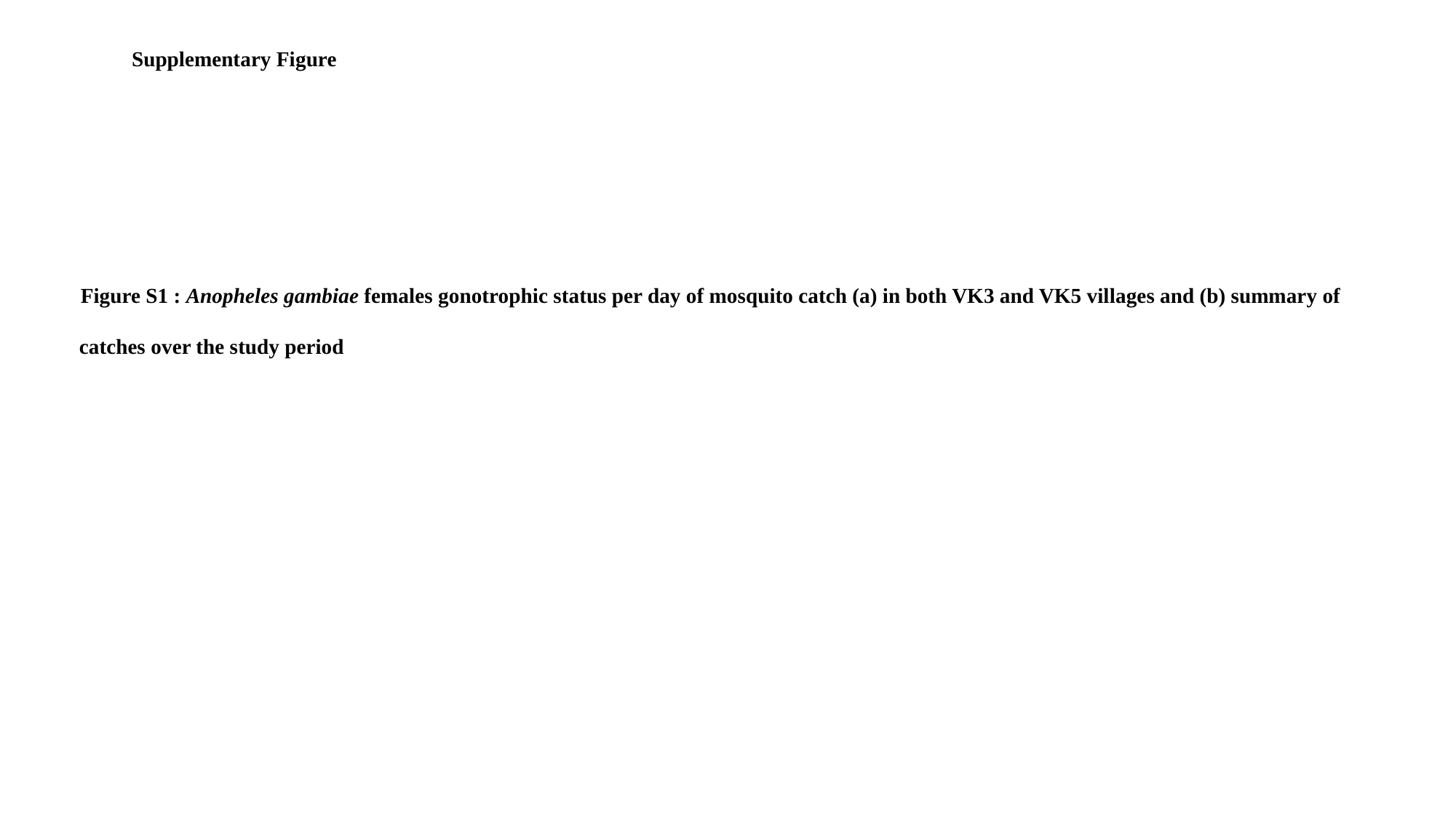

Supplementary Figure
Figure S1 : Anopheles gambiae females gonotrophic status per day of mosquito catch (a) in both VK3 and VK5 villages and (b) summary of catches over the study period

## Slide 2
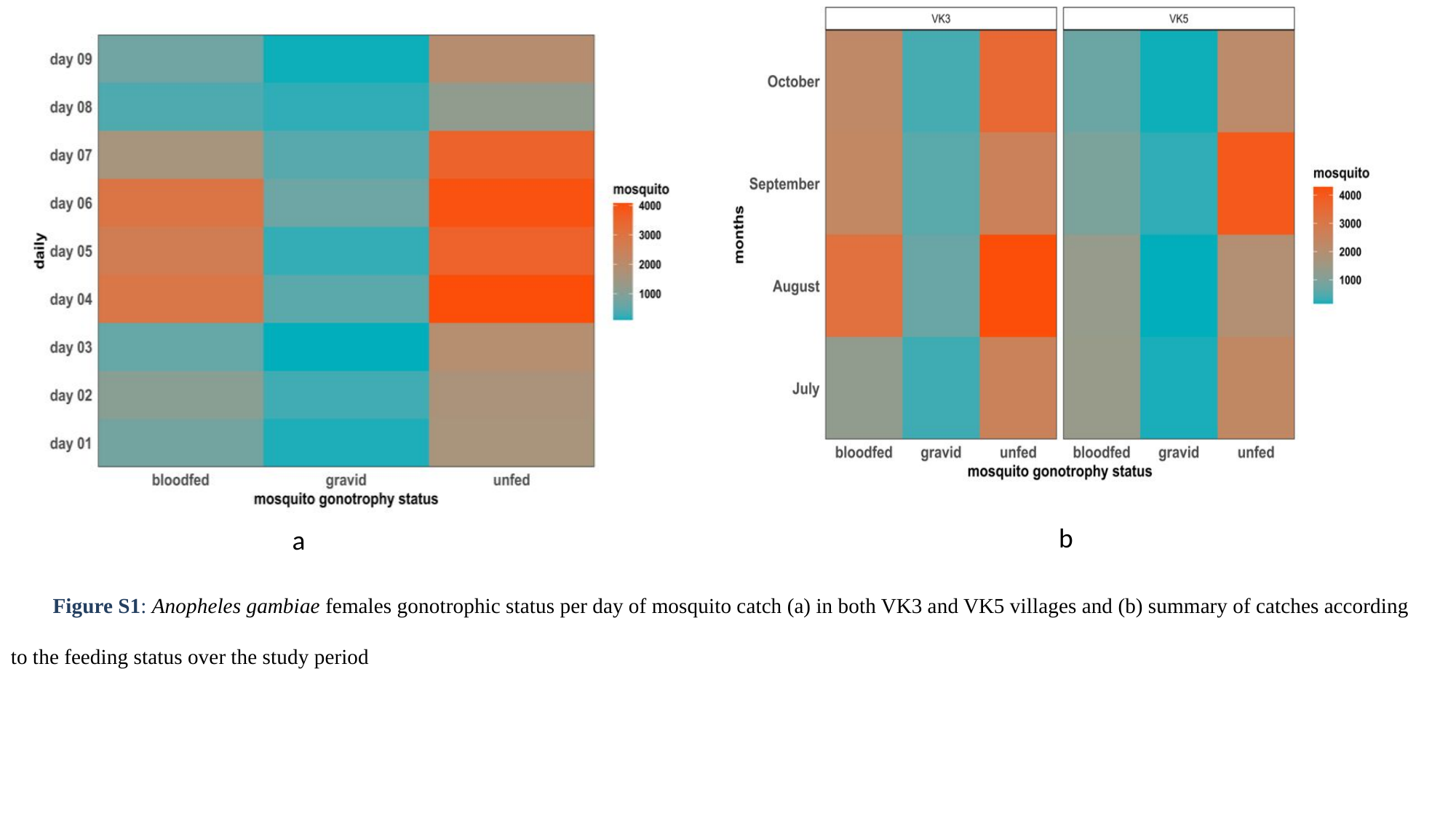

b
a
Figure S1: Anopheles gambiae females gonotrophic status per day of mosquito catch (a) in both VK3 and VK5 villages and (b) summary of catches according to the feeding status over the study period
